# Older people in custody in a forensic psychiatric facility, prevalence of dementia, and community reintegration needs: an exploratory analysis

**DOI:** 10.1186/s40352-022-00168-8

**Published:** 2022-01-24

**Authors:** Bryce E. Stoliker, Ashmini G. Kerodal, Lisa M. Jewell, Kelsey Brown, Arlene Kent-Wilkinson, Shelley Peacock, Megan E. O’Connell, J. Stephen Wormith

**Affiliations:** 1 Centre for Forensic Behavioural Science and Justice Studies, University of Saskatchewan, Saskatoon, Saskatchewan S7N 5A5 Canada; 2 Department of Psychology, University of Saskatchewan, Saskatoon, Saskatchewan S7N 5A5 Canada; 3 College of Nursing, University of Saskatchewan, Saskatoon, Saskatchewan S7N 5E5 Canada

**Keywords:** Dementia, Older adults, Forensic psychiatric hospital, Community reintegration, Health care, Social care

## Abstract

**Background:**

Across much of the developed world, the number of older people in custody has been increasing, which presents challenges for correctional systems due to the complex social, medical and mental health needs of this subgroup, especially those living with dementia. The present study therefore aimed to increase insight into the extent to which older people in custody are (a) potentially living with dementia and (b) receiving appropriate supports/services (particularly, with respect to community reintegration).

**Results:**

Cross-sectional data were drawn from a sample of 29 older people in custody and 20 correctional health care professionals at a regional forensic psychiatric hospital in a medium-sized Canadian city. In general, analyses revealed that: (a) scores from a modified version of the Community Screening Instrument for Dementia (CSI‘D’) suggest that 45% of older individuals screened positive for dementia; (b) 35% of Social Workers and 25% of Primary Nurses (i.e., RNs/RPNs) suspected that at least one older individual on their caseload has dementia, and there was adequate agreement between health staffs’ perception of the presence or absence of dementia and the CSI‘D’ assessment; (c) varying supports/services may be required for older individuals’ successful community reintegration and living; and (d) Social Workers and Primary Nurses generally lack training/education to adequately support older people in custody.

**Conclusions:**

A substantial number of older people in custody may experience age-related challenges, including dementia. This necessitates the development and implementation of programming to effectively address older individuals’ needs during incarceration and community reintegration and living.

## Background

Although correctional facilities predominantly comprise of younger individuals, the number of older people in custody has been rapidly increasing (Kakoullis et al., 2010; see also Brooke et al., 2020; Maschi et al., 2012). This is often referred to as the aging, or ‘graying’, of correctional populations, which has been documented in several countries, including Canada (Barry et al., 2017; Blowers & Blevins, 2015; Brooke et al., 2020; Maschi et al., 2012; McKendy et al., 2019; Stoliker & Galli, 2019; Uzoaba, 1998). In general, the increasing number of older people in custody has been linked to population aging, more crimes and arrests later in life, and strict sentencing legislation (see Barry et al., 2017; Blowers & Blevins, 2015; Luallen & Cutler, 2017; Regan et al., 2003; Uzoaba, 1998). Alongside the trend of an aging correctional population, evidence suggests that a notable proportion of older people in custody are living with one or more psychiatric issues (see Stoliker & Galli, 2019) which may include neurocognitive issues, such as dementia (Brooke et al., 2020; Maschi et al., 2012; Peacock et al., 2019). In addition, older people in custody show high rates of physiological issues and complex health needs (see Colsher et al., 1992; Fazel et al., 2001a, 2001b; Kakoullis et al., 2010; Lawson, 2014). Despite this understanding, there have been limited empirical investigations into the social, medical and mental health needs of older people in custody in general (Fazel et al., 2001a, 2001b, 2004; Koenig et al., 1995; Stoliker & Galli, 2019; see also Michel et al., 2012), and specifically within the context of dementia (Brooke et al., 2020; Peacock et al., 2019), as well as the extent to which these needs are being met.

### Defining ‘older’ people in custody

What qualifies an individual as ‘older’ or ‘aging’ undoubtedly varies across context and population (Uzoaba, 1998). At present, there has yet to be full consensus as to what age-threshold ought to be used to distinguish younger from older people in custody (Merkt et al., 2020). However, most commonly, older people in custody have been classified as aged 50 years and older (Brooke et al., 2020; Grant, 1999; Horowitz, 2013; Morton, 1992; Opitz-Welke et al., 2019; Stoliker & Galli, 2019; Uzoaba, 1998). The use of such a low age-threshold for studying people in custody is primarily justified in the fact that these individuals often display an ‘accelerated’ physical age, experiencing physiological illness earlier in the lifespan than persons who are not incarcerated (Barry et al., 2017; Grant, 1999; Kratcoski & Pownall, 1989; Maschi et al., 2013; Reviere & Young, 2004; Williams et al., 2006). A shortcoming of this classification, however, is that it does not recognize racial and ethnic disparities in health. For instance, it is preferable to classify older Indigenous criminal justice clients as aged 45 years and older considering the onset of health issues, including dementia, may occur at a younger age for this demographic (Baidawi et al., 2011; Brooke & Rybacka, 2020; du Toit et al., 2019; Hendrie et al., 1993; Jacklin et al., 2013; MacDonald et al., 2018). Therefore, for the purposes of this study, ‘older’ people in custody are defined as any non-Indigenous individual aged 50 years and older or any Indigenous individual aged 45 years and older.

### Age-related challenges among people in custody

Dementia, a condition associated with the aging process (but not a natural part of aging), is characterized by progressive deterioration of cognitive and functional abilities that affect daily life (Alzheimer Society of Canada, 2018; Public Health Agency of Canada, 2017). Symptoms of dementia often include impairments in judgment, reasoning, and language and communication, as well as changes in personality, erratic mood and behaviour, mobility issues, loss of short- and long-term memory, and confusion (Alzheimer Society of Canada, 2018; Feczko, 2014; Public Health Agency of Canada, 2017; World Health Organization, 2020). People in custody may be at increased risk of developing dementia given the varied health and social challenges experienced by this population which are potentially linked to cognitive and functional decline, including accelerated aging, unhealthy lifestyles (e.g., poor diet, inactivity, smoking, substance use), traumatic brain injuries, psychiatric morbidities, and lower educational attainment (Baidawi et al., 2011; Brooke et al., 2020; Combalbert et al., 2018; du Toit et al., 2019; Gaston, 2018; Maschi et al., 2012; Skarupski et al., 2018; Williams et al., 2012).

Despite the elevated risk of dementia among people in custody, there is a general lack of information on the prevalence of this health problem in correctional settings. However, current estimates suggest that anywhere between 1% to 44% of older people in custody may be living with dementia depending on the nature and size of the correctional setting (Combalbert et al., 2018; Fazel et al., 2001a; Kingston et al., 2011; Maschi et al., 2012; Regan et al., 2003; Shepherd et al., 2017).^1^ For instance, among older individuals drawn from seven French prisons, approximately 20% screened positive for dementia (Combalbert et al., 2018), while US data suggest dementia rates between 1% to 30% (Skarupski et al., 2018; see also Regan et al., 2003) or 1% to 44% (Maschi et al., 2012). In addition, one UK study suggests 1% of older people in custody had dementia (Fazel et al., 2001a), whereas another identified signs of cognitive impairment in 13% of their sample of older individuals (Kingston et al., 2011).

While correctional facilities generally have policies and practices in place to assess, diagnose, and treat physical and mental health issues, the correctional setting is hardly conducive to supporting older individuals with increased and complex health issues as many facilities lack geriatric- or dementia-specific health care services (Brooke et al., 2020; Maschi et al., 2012; Peacock et al., 2019). Indeed, research has indicated that older people in custody show high rates of physical and mental health issues, yet a notable proportion do not receive adequate care for these issues during incarceration (see Stoliker & Galli, 2019). Though knowledge is limited on the nature and quality of supports and services provided to incarcerated older adults during community reintegration and living, current efforts may be insufficient in this area as well. In this case, aging individuals involved with the criminal justice system may face a variety of barriers which are not adequately addressed during community reintegration and living, including complex physical and mental health conditions, limited access to housing and long-term care, lack of support (e.g., social, medical, financial) and employable skills, as well as a stigma surrounding criminal history which further complicates these barriers (Colibaba, 2019). Accordingly, further research is required to better understand the needs of incarcerated older adults regarding community reintegration and living—especially within the Canadian context.

### The current study

With the trend towards an aging correctional population, coupled with the potential risk of developing dementia and other age-related challenges among older people in custody, there is an undeniable need for correctional services to develop and implement programming to promote the well-being of this vulnerable subgroup (Brooke et al., 2020; Maschi et al., 2012; Peacock et al., 2019; see also Colibaba, 2019). For instance, studies suggest that correctional settings should aim to improve the quality of care for people living with dementia through the implementation of appropriate cognitive screening tools, a multi-disciplinary treatment model, specialized training and support for staff, and high-quality services (Brooke et al., 2020; Peacock et al., 2019). Further, it has been suggested that successful community reintegration of aging individuals involved with the criminal justice system may be achieved through (a) improved practices for discharge planning which aim to increase access to community supports, and (b) educating health care professionals on the risks and needs experienced by this population (Colibaba, 2019). To address the need to improve the quality of care and community reintegration practices concerning older people in custody with age-related challenges, the current study sought to investigate the extent to which: (1) older individuals screen positive for dementia; (2) Social Workers (SW) and Primary Nurses (PN) perceive older individuals under their mutual care to have dementia; (3) older individuals require certain supports/services for successful community reintegration; and (4) SWs and PNs are provided training to accommodate older individuals.

## Method

### Setting and participants

Between April and August 2019, a cross-sectional study was conducted to assess dementia and other age-related needs of older individuals at a regional forensic psychiatric hospital located in a medium-sized Canadian city. This hospital provides intensive psychiatric services to men and women involved in the federal or provincial justice systems and has approximately 345 staff members and a capacity of 171 forensic patients. Participants included 29 older people in custody and 20 correctional health care professionals (i.e., 8 SWs and 12 PNs [Registered Nurses or Registered Psychiatric Nurses]) with older individuals on their caseloads. For older people in custody, participants’ ages ranged from 46 to 80 years (*M* = 59.30; *SD* = 8.67), 3.4% identified as female, and 55.2% reported Indigenous status. For correctional health care professionals, participants’ ages ranged from 22 to 58 years (*M* = 37.25; *SD* = 11.36), 90% identified as female, and 10% reported Indigenous status. In addition, these health staff members had worked at the facility for a range of 1 to 23 years (*M* = 9.21; *SD* = 7.01), 80% had held their current position for over 2 years, and there were between 1 to 21 older individuals on their caseloads (*M* = 4.67; *SD* = 5.97).

### Sampling and data collection

A two-stage sampling and data collection procedure was adopted. In the first stage, an up-to-date list of all older individuals (i.e., any non-Indigenous individual aged 50 years and older or any Indigenous individual aged 45 years and older) residing in the facility was extracted from Correctional Service Canada’s Offender Management System (OMS) in February 2019. Of the 55 older individuals residing in the facility, a total of 29 were screened for dementia. Individuals were excluded from screening if they were deemed to be dangerous (*n* = 2); had a limited capacity to provide consent (*n* = 1); for medical reasons (*n* = 1); were discharged or transferred to another facility prior to the data collection period (*n* = 7); or declined participation (*n* = 15). In the second stage, correctional health care professionals (i.e., SWs and PNs) were selected to complete self-report surveys pertaining to older individuals on their caseloads. Specifically, SWs (*N* = 8) and PNs (*N* = 18) were selected if (a) they had an individual on their caseload who had been screened for dementia and (b) that individual consented to having their SW and/or PN share their information with the research team. SWs and PNs received self-report surveys for each individual on their caseload who had been screened for dementia. Each survey was identical and consisted of items pertaining to patients’ physical and mental health status, involvement in institutional programming, and discharge planning and needs. SWs and PNs were also asked about training with respect to working with older people in custody. All SWs (*n* = 8) and 67% of PNs (*n* = 12) responded to the survey. To protect the identity of participants, all health staff originally selected for participation (*N* = 26) were instructed to return the survey package to a member of the research team irrespective of whether surveys were completed.

### Measures

#### Dementia

The Community Screening Instrument for Dementia (CSI‘D’; Hall et al., 1993, 1996, 2000; Hendrie et al., 1995; Unverzagt et al., 1999) was used to identify, among the subset of 29 older individuals residing in the facility who agreed to participate in the study, those at risk for dementia and who require further clinical assessment.^2^ The CSI‘D’ contains a Cognitive Score obtained from a participant interview and an Informant Score obtained from an informant (i.e., caregiver) questionnaire (Hall et al., 1993, 1996, 2000).^3^ The Cognitive Score and Informant Score are then combined to create the Discriminant Score, which signifies risk for dementia (Hall et al., 1996, 2000). For the current study, the Discriminant Score was computed from the combination of the Cognitive Score from the interview with older individuals and Informant Score from the PNs’ survey. A separate scoring protocol for the Discriminant Score was used for Indigenous participants (0.461839 - [0.012164 * Cognitive Score] + [0.045880 * Informant Score]) and non-Indigenous participants (0.564786 - [0.015019 * Cognitive Score] + [0.044918 * Informant Score]), as it has been found to mitigate educational and cultural biases (Hall et al., 2000, p. 526) and was recommended by one of the tool developers as appropriate for this study (Hugh C. Hendrie, personal communication, September 18, 2019). In the event that an Informant Score is not available, the Cognitive Score can be used on its own to identify risk for dementia (however, accuracy is maximized when both scores are used).

Older individuals were placed into the following categories based on the Discriminant Score (DS), or the Cognitive Score (CS) when the Informant Score was unavailable: good performance = DS < 0.120 or CS only > 29.5; intermediate performance = DS 0.120–0.183 or CS only > 28.5 ≤ 29.5; poor performance = DS ≥ 0.184 or CS only ≤28.5. Those with poor performance were flagged for a clinical dementia assessment (Hall et al., 1996, p. 136). Furthermore, to assess health staff perception of whether older individuals have dementia, SWs and PNs were asked how likely it is that each individual on their caseload has dementia (yes/no).^4^

#### Discharge planning/community reintegration

Several items captured older individuals’ discharge needs. In particular, SWs and PNs were asked whether each older individual on their caseload has any chronic illnesses, cognitive limitations, physical limitations, and mental health challenges “… which should be considered in discharge planning.” SWs and PNs were also asked how important or unimportant various supports/services were for successful community reintegration of each older individual on their caseload, including: transitional housing (e.g., halfway house, emergency shelter, etc.); social housing (e.g., low income); permanent supportive housing (e.g., senior’s homes, care homes, etc.); employment (e.g., help finding a job); financial (e.g., social assistance); health (e.g., primary care, care for chronic illnesses, etc.); end of life care (e.g., palliative care); medical benefits (e.g., for prescription medication); medication administration (e.g., outpatient clinic, support worker, etc.); mental health (e.g., counselling); psychiatric (e.g., diagnosis, medications, etc.); addiction support; cognitive supports/services (e.g., disabilities, FASD, etc.); mobility (e.g., physical disabilities); caregiver for offender; family reunification support; cultural (e.g., church, Elders, etc.); legal (e.g., power of attorney, will, etc.); and meal support (e.g., meals on wheels). All discharge/community reintegration items were binary coded (yes/no).

#### Health staff training

Several items captured correctional health care professionals’ perceptions of training needs to adequately manage older individuals residing in the facility. Specifically, SWs and PNs were asked if they had received: (i) specialized training in recognizing and responding to dementia in older people in custody; (ii) any other specialized training about older people in custody (excluding dementia training); and (iii) adequate training/education to support the older individuals on their caseload. Each item was binary coded (yes/no).

### Analytic procedure

The Statistical Package for the Social Sciences (SPSS) version 25 was used for all data analyses. Foremost, CSI‘D’ scores were calculated, which were used to categorize older individuals according to performance level (i.e., good, intermediate, or poor performance) and, subsequently, to estimate prevalence rates for risk of dementia (i.e., a positive screen and recommendation for further clinical assessment). Prevalence rates were also estimated for health staffs’ perception of the likelihood that older individuals on their caseload have dementia, separately for SWs and PNs. A similar approach was used to estimate prevalence rates for health staffs’ perception of factors that should be considered in older individuals’ discharge planning, as well as staff perception of supports/services deemed important for older individuals’ successful community reintegration.

Tests of interrater reliability were performed to assess the extent to which SWs and PNs agree on the likelihood of the presence of dementia, discharge needs, and importance of community reintegration supports/services for older individuals under their mutual care (i.e., comparing responses between these correctional health care professionals across each older individual). Specifically, agreement between SWs and PNs was assessed to provide some insight into the level of consistency across these health care professionals with respect to the provision of health care services—a greater level of agreement on older individuals’ needs would suggest a greater level of consistency. Following McHugh’s (2012) recommendations for health research, percent agreement and Cohen’s kappa statistic, *κ*, were used to measure agreement between SWs and PNs on the abovementioned domains. Percent agreement is directly interpreted as the percent of cases (i.e., older individuals) that raters (i.e., SWs and PNs) achieved agreement for a particular variable. Interpretation of *κ* is based on McHugh’s (2012) rules for health data, whereby values ≤0–0.20 indicate no agreement, 0.21–0.39 as minimal, 0.40–0.59 as weak, 0.60–0.79 as moderate, 0.80–0.90 as strong, and > 0.90 as almost perfect agreement. It is recommended that 80% should be the minimum acceptable percent agreement, whereas *κ* below 0.60 indicates inadequate agreement among the raters (McHugh, 2012). Finally, prevalence rates were estimated for health staffs’ perception of training needs.

Overall, relatively few cases were missing and, therefore, pairwise deletion was used when estimating descriptive and inferential statistics. With respect to hypothesis testing, probability values < 0.05 were considered statistically significant.

## Results

Among the subset of 29 older individuals residing in the facility who were screened for dementia, Discriminant Scores (or Cognitive Scores) from the CSI‘D’ suggest that 45% (*n* = 13) of patients had poor performance (i.e., a positive screen and potential risk for dementia) and, therefore, meet the criteria for a clinical dementia assessment. Among the 20 correctional health care professionals who participated in this study, 35% (*n* = 7) of SWs and 25% (*n* = 5) of PNs reported that at least one older individual on their caseload likely has dementia. With respect to the extent to which SWs and PNs agree on the likelihood of dementia among older individuals under their mutual care, tests of interrater reliability suggest adequate agreement (*N* = 20; percent agreement = 90%; *κ* = 0.765, *p* < .001). It is also worth noting there was adequate agreement between health staffs’ perception of the presence or absence of dementia among older individuals on their caseload and the CSI‘D’ assessment (*N* = 29; percent agreement = 83%; *κ* = 0.644, *p* < .001).

With respect to health staffs’ perception of factors that should be considered in older individuals’ discharge planning (Table 1), a majority of SWs and PNs reported that at least one older individual on their caseload has a chronic illness (65% and 80%, respectively), cognitive limitations (65% and 65%), physical limitations (55% and 55%), and mental health challenges (60% and 55%). However, tests of interrater reliability suggest there was inadequate agreement between SWs and PNs on discharge needs of older individuals under their mutual care (see Table 1).

**Table 1:** Health staffs’ perception of factors that should be considered in older individuals’ discharge planning (*N* = 20)

Discharge Needs	Social Worker % (*n*)	Primary Nurse % (*n*)	% Agreement	*κ*	*p*-value
Chronic illness	65 (13)	80 (16)	65	0.146	.482
Cognitive limitations	65 (13)	65 (13)	70	0.341	.128
Physical limitations	55 (11)	55 (11)	70	0.394	.078
Mental health challenges	60 (12)	55 (11)	65	0.286	.199

Furthermore, Table 2 presents results from analyses on health staffs’ perceptions of supports/services deemed important for older individuals’ successful community reintegration. A majority of SWs and PNs reported that at least one older individual on their caseload would benefit from transitional housing (69% and 75%, respectively), permanent supportive housing (75% and 62%), employment support/services (56% and 62%), financial support/services (81% and 100%), health services (94% and 100%), medical benefits (100% and 100%), medication administration (88% and 100%), mental health support/services (94% and 100%), psychiatric services (88% and 94%), addiction support (75% and 56%), family reunification (81% and 75%), and cultural support/services (75% and 50%). Alternatively, a lower proportion of SWs and PNs reported that at least one older individual on their caseload would benefit from end-of-life care (19% and 25%, respectively), cognitive support (31% and 44%), mobility support/services (38% and 44%), and a caregiver (50% and 38%). A larger proportion of PNs compared with SWs reported that at least one older individual on their caseload would benefit from social housing (69% and 44%, respectively) and legal support/services (69% and 44%), whereas a larger proportion of SWs compared with PNs reported that at least one older individual on their caseload would benefit from meal support (94% and 44%, respectively). Tests of interrater reliability suggest adequate agreement between SWs and PNs on the importance of the following supports/services for successful community reintegration of older individuals under their mutual care: employment support/services (percent agreement = 81%; *κ* = 0.613, *p* < .05); psychiatric services (percent agreement = 94%; *κ* = 0.636, *p* < .01); addiction support (percent agreement = 81%; *κ* = 0.600, *p* < .01); financial support/services (percent agreement = 81%); health services (percent agreement = 94%); end-of-life care (percent agreement = 81%); medical benefits (percent agreement = 100%); medication administration (percent agreement = 88%); and mental health support/services (percent agreement = 94%).

**Table 2:** Health staffs’ perception of supports/services that are important for older individuals’ successful community reintegration (*N* = 16)

Supports/Services	Social Worker % (*n*)	Primary Nurse % (*n*)	% Agreement	*κ*	*p*-value
Transitional housing	69 (11)	75 (12)	69	0.231	.350
Social housing	44 (7)	69 (11)	50	0.045	.838
Permanent supportive housing	75 (12)	62 (10)	63	0.143	.551
Employment	56 (9)	62 (10)	81	0.613	.013
Financial^a^	81 (13)	100 (16)	81		
Health^a^	94 (15)	100 (16)	94		
End of life care	19 (3)	25 (4)	81	0.455	.064
Medical benefits^a^	100 (16)	100 (16)	100		
Medication administration^a^	88 (14)	100 (16)	88		
Mental health^a^	94 (15)	100 (16)	94		
Psychiatric	88 (14)	94 (15)	94	0.636	.006
Addiction support	75 (12)	56 (9)	81	0.600	.009
Cognitive support	31 (5)	44 (7)	63	0.213	.377
Mobility	38 (6)	44 (7)	69	0.355	.152
Caregiver	50 (8)	38 (6)	50	0.000	1.000
Family reunification	81 (13)	75 (12)	56	−0.273	.267
Cultural	75 (12)	50 (8)	63	0.250	.248
Legal	44 (7)	69 (11)	50	0.045	.838
Meal support	94 (15)	44 (7)	50	0.099	.362

^a^Measures of association could not be computed because correctional health care providers’ perceptions were a constant

Finally, among the 20 correctional health care professionals who participated in this study, 95% (*n* = 19) reported they had not received specialized training in recognizing and responding to dementia in older people in custody. In addition, 100% (*n* = 20) reported they had not received any other specialized training about older people in custody (excluding dementia training). It was also found that 60% (*n* = 12) reported they had not received adequate training/education to support the older individuals on their caseload.

## Discussion

Across much of the developed world the number of older people in custody has been increasing, which presents challenges for correctional systems due to the complex needs of this subgroup. Still, there is limited knowledge on the social, medical and mental health needs of older people in custody and the extent to which these needs are being met (see Stoliker & Galli, 2019; see also Michel et al., 2012), especially concerning cognitive impairment, dementia and dementia-related conditions (Brooke et al., 2020; Maschi et al., 2012; Peacock et al., 2019). The present study aimed to address this gap, investigating in greater detail the extent to which older people in custody in a forensic psychiatric facility are (a) potentially living with dementia and (b) receiving appropriate supports/services (particularly, with respect to community reintegration). While our study is exploratory and results are preliminary, several important implications can be extracted from this research.

Several researchers have emphasized the necessity of incorporating cognitive screening tools into correctional policy and practice, suggesting these assessments be administered at admission and on an annual basis to improve detection of dementia-related conditions among older people in custody (Brooke et al., 2020; Peacock et al., 2019). While studies in the correctional setting have adopted various instruments to identify the prevalence of cognitive impairment and dementia among older people in custody (Combalbert et al., 2018; Fazel et al., 2001a; Kingston et al., 2011; Regan et al., 2003; Shepherd et al., 2017), there is still a need for an optimum cognitive screening tool specific to the correctional context (Brook et al., 2020). The current study utilized the Community Screening Instrument for Dementia (CSI‘D’), which had not yet been used with correctional populations but has been validated in a diverse set of community samples (Davoudkhani et al., 2019; Hall et al., 1993, 1996; Hendrie et al., 1995; Unverzagt et al., 1999).

Based on the CSI‘D’ assessment, nearly half (45%) of the individuals screened for dementia met the criteria for further clinical assessment (i.e., a positive screen and potential risk for dementia). This is slightly greater than the upper limit of the range obtained from a meta-analysis of dementia studies conducted in U.S. correctional settings (1% - 44%: Maschi et al., 2012). This finding suggests that dementia-related conditions may be a relevant issue for older people in custody in forensic psychiatric hospitals. Though, it is plausible this may be an overestimation of the true risk of dementia. Indeed, health screens such as the CSI‘D’ tend to be overinclusive to ensure persons in need receive health services (Trevethan, 2017); thus, it is likely that rates of diagnosed dementia would be more conservative. It has also been argued that current cognitive screening tools are unsuitable for identifying cases of dementia among those residing in custodial settings as these tools are not often tailored for the correctional context (Brook et al., 2020). For this reason, the CSI‘D’ was modified (in consultation with the original tool developers) to more appropriately assess individuals in a correctional setting. Despite this effort, the reliability and validity of this modified version of the CSI‘D’ is unknown, as the modifications could introduce Type I or II error. At any rate, researchers must prioritize developing and/or validating culturally sensitive cognitive screening tools for correctional settings to accurately detect suspected cases of dementia and, subsequently, provide these individuals with specialized support.

In addition to incorporating optimum cognitive screening tools into correctional policy and practice, correctional health care professionals and correctional officers would benefit from specialized training to accurately identify and support older people in custody living with cognitive impairment or dementia-related conditions (Brooke et al., 2020; Peacock et al., 2019). This has important implications as a lack of training may have an impact on older individuals’ quality of life and access to appropriate health services during incarceration, as well as during community reintegration and living. Findings from the current study suggest that 25% to 35% of correctional health care professionals (i.e., PNs and SWs, respectively) suspected that at least one older individual on their caseload has dementia. It was also found there was adequate agreement between health staffs’ perception of the presence or absence of dementia and the CSI‘D’ assessment. This suggests that correctional health care professionals were able to recognize dementia-related conditions among older people in custody. Interestingly, however, nearly all SWs and PNs (95%) reported they had not received specialized training in recognizing and responding to dementia in older people in custody. Therefore, while there were consistencies between health staffs’ perception and CSI‘D’ classification of risk for dementia, there is limited reliability and validity in these findings as a lack of proper training precludes one’s ability to accurately recognize and respond to dementia-related conditions. Furthermore, all SWs and PNs reported they had not received any other specialized training about older people in custody (i.e., excluding dementia training) and nearly two-thirds believed they had not received adequate training to support the older individuals on their caseload.

Accordingly, there is an urgent need to increase training initiatives for correctional health care professionals and likely all correctional personnel (e.g., correctional officers, parole officers) to better support older people in custody, especially those with complex age-related problems such as dementia. While training programs have been developed to improve correctional staffs’ (and even peer) response to the needs of older people in custody, the impact of existing programs is not fully understood (see Brooke et al., 2020). Correctional administrators and researchers should therefore prioritize the development and evaluation of training programs aimed to enhance the support of older individuals in custody. Furthermore, although findings from the current study suggest that SWs and PNs agree on several discharge and community reintegration needs of older individuals on their caseloads, there was not always a high level of agreement about the supports required by older individuals. Standardized training about older individuals’ needs and how to address them could lead to greater consistency amongst correctional personnel and, ultimately, better overall provision of health care services. In this case, correctional settings could benefit from training which promotes a coordinated approach to medical and social care of older people in custody, with increased sharing of information and communication (Brooke et al., 2020; Peacock et al., 2019). Of particular importance would be the introduction of high-quality geriatric medicine into correctional settings as geriatricians are trained to promote physical and cognitive functioning, as well as focus on better overall well-being as a goal (Stoliker et al., 2020). A challenge, however, is the low number of geriatricians available to the general public and the difficulty of attracting these specialists into correctional medical systems.

Importantly, there has been little scholarly or public attention directed toward reintegration of older adults into the community upon release from custody (Colibaba, 2019). This is surprising considering the challenges these individuals may face with respect to community reintegration and living. For instance, age-related physical and mental health conditions, as well as a disruption in financial and social stability due to incarceration, may introduce barriers to employment, medical care, and housing (see Colibaba, 2019). This may be exacerbated by the fact that correctional facilities generally lack specialized geriatric programming to meet older individuals’ needs during incarceration (Brooke et al., 2020; Maschi et al., 2012; Peacock et al., 2019) and during community reintegration and living (Colibaba, 2019).

With respect to the current study, a majority of correctional health care professionals (i.e., SWs and PNs) indicated that chronic illness, cognitive limitations, physical limitations, and mental health challenges should be considered in discharge planning. Many also highlighted that successful community reintegration may depend on housing support, employment and financial support, physical and mental health services, addiction support, family reunification, cultural services, legal support, and meal support. A lower, yet noteworthy, proportion reported that older individuals would benefit from end-of-life care, cognitive support, mobility support/services, and a caregiver. Therefore, older adults transitioning into the community upon release from custody will require a wide range of supports/services. A challenge to the current data, however, is that we were unable to delineate whether these factors have been (or will be) included in older individuals’ discharge planning and whether they will be connected to appropriate supports/services upon release from custody. While researchers and policymakers have highlighted strategies to improve community reintegration of incarcerated older adults (see Colibaba, 2019), more research is needed on the nature and quality of discharge planning and initiatives to accommodate these individuals while in the community. In this case, researchers should incorporate the voices of older people in custody to better understand barriers and potential solutions for successful community reintegration and living (Colibaba, 2019).

### Limitations

Findings should be interpreted in light of several limitations. First, the low response rate among older individuals may have impacted the reliability of results for the CSI‘D’. Nearly half of prospective participants were ultimately unable to participate (20%) or declined to participate (27%) and, among those who declined to participate, many cited “no issues or problems with memory/dementia” as the reason. Second, the modified version of the CSI‘D’ used in the current study is not a diagnostic instrument—diagnosing dementia is complex and takes an interdisciplinary team. In addition, this cognitive screening tool has yet to be rigorously assessed for reliability and validity. This further confounds the true rate of dementia within the current sample. Despite this limitation, it is apparent that concerns about dementia risk exist in corrections and, therefore, future research will be necessary to cross-validate this cognitive screening tool against dementia diagnoses derived from a thorough clinical evaluation. This is the focus of the next phase of our research, as the goal is to develop a standardized, reliable, and valid instrument for dementia assessment in (Canadian) correctional populations. Third, the survey completed by SWs and PNs relied upon retrospective self-reports, which is vulnerable to biased recall and social desirability. It is possible that SWs’ and PNs’ knowledge of the study influenced responses related to dementia, as well as perceptions of supports/services necessary for discharge planning and community reintegration. Fourth, the regional forensic psychiatric hospital that participated in the current study is a unique and specialized setting, which houses a high rate of people with complex mental health needs, older individuals, and Indigenous people. Relatedly, the prevalence of neurocognitive (and other psychiatric) illnesses, as well as the availability of healthcare resources, may not be comparable to other (more general) correctional settings. Therefore, current findings may have limited generalizability and should be considered within the context of the correctional setting for this study. Generalizability may also be limited due to the small sample size; thus, future research should consider including larger and more diverse samples of older people in custody and correctional health care professionals to investigate the current research objectives.

## Conclusion

With the trend towards an aging correctional population, it is imperative to develop and improve programming which aims to address the social, medical and mental health needs of older people in custody (especially those living with cognitive impairment, dementia and dementia-related conditions). This may include the implementation of effective screening practices, specialized training for staff to support older individuals, a multi-disciplinary and collaborative approach to treatment, as well as specialized geriatric programming to meet older individuals’ needs during custody and community reintegration and living. While the current study offers preliminary insights, further research is necessary to address significant gaps in knowledge. Most notably, researchers have yet to identify an optimum cognitive screening tool for correctional settings, and there is a profound lack of understanding of institutional and community programming for older people in contact with the criminal justice system.

## Data Availability

The dataset generated and/or analyzed during the current study is not publicly available because it contains personally identifying information. Informed consent was not obtained for publication of the dataset due to the small sample size which prohibited the researchers’ ability to protect the anonymity and confidentiality of the participants.

## Footnotes

1 These estimates are derived from dementia screening tools and, because these screening tools have not been validated for correctional settings, error rates (i.e., false positives and false negatives) are unknown.

2 The CSI‘D’ was selected for the current study as it is a culturally sensitive tool that includes scoring protocols for Indigenous and non-Indigenous persons (i.e., the dominant populations in the current study). In fact, the version of the CSI‘D’ employed was developed in Canada (in consultation with Elders in Manitoba) and has been validated in racially and ethnically diverse samples to mitigate issues of cultural bias. Details on the empirical characteristics of the CSI‘D’, as assessed through the current research, can be found in Kerodal et al. (2020).

3 While the CSI‘D’ provides a valid flag for high-risk Indigenous and non-Indigenous persons in the community in need of a more thorough clinical dementia assessment, it contains items that are not valid for people in custody. Accordingly, the authors modified these items to adequately assess those residing in a custodial setting. The modified CSI‘D’ participant interview contained items on memory (4), abstract thinking (4), higher cortical function (10), praxis (5), orientation to time (5), and orientation to place (5), which were used to produce the Cognitive Score (range: 0–33; perfect score = 33). The modified CSI‘D’ informant questionnaire contained items on memory and cognition (11), activities of daily living (7), and miscellaneous problems (5), which were used to produce the Informant Score (range: 0–30; perfect score = 0). A detailed description of items from the modified CSI‘D’ participant interview and informant questionnaire, as well as scoring protocol, can be found in Kerodal et al. (2020).

4 Health staffs’ perception of whether older individuals have dementia is measured separately from the CSI‘D’ assessment (i.e., the modified informant questionnaire).
